# Phosphate in Virulence of *Candida albicans* and *Candida glabrata*

**DOI:** 10.3390/jof6020040

**Published:** 2020-03-26

**Authors:** Julia R. Köhler, Maikel Acosta-Zaldívar, Wanjun Qi

**Affiliations:** Division of Infectious Diseases, Boston Children’s Hospital/Harvard Medical School, Boston, MA 02115, USA; Maikel.AcostaZaldivar@childrens.harvard.edu (M.A.-Z.); Wanjun.Qi@childrens.harvard.edu (W.Q.)

**Keywords:** PHO regulon, Target of Rapamycin, virulence, oxidative stress resistance, cell wall, metabolomics, antifungal, drug target

## Abstract

*Candida* species are the most commonly isolated invasive human fungal pathogens. A role for phosphate acquisition in their growth, resistance against host immune cells, and tolerance of important antifungal medications is becoming apparent. Phosphorus is an essential element in vital components of the cell, including chromosomes and ribosomes. Producing the energy currency of the cell, ATP, requires abundant inorganic phosphate. A comparison of the network of regulators and effectors that controls phosphate acquisition and intracellular distribution, the PHO regulon, between the model yeast *Saccharomyces cerevisiae*, a plant saprobe, its evolutionarily close relative *C. glabrata*, and the more distantly related *C. albicans*, highlights the need to coordinate phosphate homeostasis with adenylate biosynthesis for ATP production. It also suggests that fungi that cope with phosphate starvation as they invade host tissues, may link phosphate acquisition to stress responses as an efficient mechanism of anticipatory regulation. Recent work indicates that connections among the PHO regulon, Target of Rapamycin Complex 1 signaling, oxidative stress management, and cell wall construction are based both in direct signaling links, and in the provision of phosphate for sufficient metabolic intermediates that are substrates in these processes. Fundamental differences in fungal and human phosphate homeostasis may offer novel drug targets.

## 1. *Candida albicans* is a Prevalent Human Commensal

This review focuses on the role of phosphate homeostasis in virulence of *Candida albicans* and *C. glabrata*, the most frequently isolated fungal species causing invasive disease in humans. Excellent reviews of the role of phosphate in virulence of other fungi have appeared [1,2] so we here focus on recent findings in *Candida*. *C. albicans,* the main focus of this review, colonizes oral and gastrointestinal mucous membranes in 40%–75% of healthy individuals [3,4,5]. It is thought to have co-evolved with its human host for millions of years [6]. But novel findings suggest that this species and its close relatives may have begun to colonize humans more recently than previously believed. It may be associated with plant environments overlooked by previous sampling efforts: recently it was isolated from bark of old-growth oak trees [7]. People in an Amazonian traditional community are rarely colonized by *C. albicans* [8]. One interpretation of these reports is that current highly prevalent human colonization states with *C. albicans* may have emerged during recent developments of human ecology like the rise of agriculture or industrialization. Nevertheless, whenever it was that *C. albicans* began its association with human mucous membranes, the subsequent large increase of its host population ensured its success as a commensal.

## 2. *C. albicans* Is Not a Highly Evolved Human Pathogen but It Can Exploit Host Weaknesses

*C. albicans* is not particularly well adapted as a human pathogen. Among the ascomycota, the phylum to which *Candida* belong, many species in the order *Onygenales* are professional pathogens with a life cycle that alternates between invasive disease of warm-blooded animals and proliferation in their victims’ cadavers and in the soil, from there producing infective propagules that can be inhaled to infect another animal. In contrast, overwhelming infection of the host is a dead end for *Candida*: It has no clear path from the soil back to its preferred niche, human mucous membranes. Until the last century, human populations susceptible to invasive candidiasis were vanishingly small. Still, *C. albicans* wields significant virulence factors that provide it access to host nutrient stores [9]. The ability to damage and digest host tissues and to withstand some host immune defenses, may have evolved to enable the fungus to spread via limited mucous membrane infections among the single human population naturally susceptible to *C. albicans*: Infants. In young infants, before development of anti-candidal adaptive immunity, thrush is common. Infants can spread sufficient inocula to their caregivers to maintain a large population of human hosts for *Candida* commensalism [10]. The virulence determinants honed by *C. albicans* against infants are sufficient to drive devastating invasive infections among today’s large immunocompromised patient populations, e.g., those undergoing chemotherapy or transplants, those requiring significant surgical interventions or depending on long-term indwelling lines like dialysis- and parenteral nutrition-dependent patients.

## 3. Phosphate is Indispensable for *C. albicans* Growth, a Precondition for its Pathogenesis

*Candida* species are among the majority of pathogens that, unlike e.g., *Clostridium tetani,* can only cause disease if they grow and replicate for many generations. During growth in human tissues, *Candida* cells must withstand host environmental conditions very unlike their growth optima: Temperatures ≥36 °C, nutrient deprivation especially for glucose and phosphate, microaerophilic or anaerobic environments, and pH 7.35–7.45 [11]. They must additionally resist host immune cells like neutrophils and macrophages that actively deploy oxidative, nitrosative and cationic stressors, as well as cell wall stress [12]. Phosphate is critical for *Candida* cells to grow, replicate and withstand host-imposed stress during invasive disease.

Phosphorus is an essential element for all cells as an abundant component of macromolecules. Without sufficient phosphate there is no cellular growth because ribosomal RNA, mRNAs, and tRNA cannot be synthesized and phospholipid membranes cannot be expanded. There is also no cell division, because DNA cannot be replicated. More fundamentally, there is no energy production: Initiation of glycolysis requires two phosphate moieties per glucose molecule, and oxidative phosphorylation depends on phosphate repletion in the mitochondrial matrix [13]. In the model yeast *Saccharomyces cerevisiae*, the mitochondrial membrane potential itself requires phosphate transport into the matrix [14].

## 4. Phosphate Acquisition Differs Fundamentally between Organisms That Ingest Food, and Those That Absorb It

Phosphate acquisition and homeostasis inevitably are core components of all cells’ regulatory systems. But the regulatory needs of absorbing organisms differ fundamentally from those that ingest their food. Organisms like amoebae and animals, that obtain their nutrition by ingesting cells, will concomitantly acquire ample phosphate together with the nitrogen- and carbon sources of which these cells are composed. In contrast, organisms that absorb their nutrients, often after extracellular digestion by secreted enzymes, like bacteria, fungi, and plants, must import phosphate against a steep concentration gradient [15]. For this reason, phosphate homeostatic systems of the latter group of organisms have many similarities despite the large evolutionary distances between them.

Phosphate homeostatic systems of bacteria and fungi, i.e. absorbing organisms, include plasma membrane phosphate transporters, phosphate sensors that may be extra- or intracellular, mechanisms to store surplus as polyphosphate, and regulatory systems including a central transcription factor to maintain phosphate repletion without intoxicating the cytoplasm with orthophosphate. These systems are called the PHO regulon and have been studied extensively in bacteria [16] and in *S. cerevisiae* [17,18,19]. In pathogenic bacteria, phosphate acquisition is known to be crucial in virulence [20,21,22]. A recent superb review compares the *S. cerevisiae* PHO regulon with those of its elements known to be conserved in *C. albicans* and other human fungal pathogens [1]. The activity of many of these elements is only beginning to be characterized, and novel components remain to be discovered, but in broad strokes, the PHO regulon of these fungi resembles that of *S. cerevisiae* [23].

## 5. Recognizing a Role of Phosphate in *C. albicans* Virulence

The role of phosphate homeostasis in *C. albicans* pathogenesis was first recognized by the Aberdeen Fungal Group who characterized and quantified virulence phenotypes of 43 clinical *C. albicans* isolates representing 4 known clades [24]. They correlated expression profiles among a subset of these strains, 5 from each clade, with virulence phenotypes. Five among 18 genes differentially expressed in isolates displaying differing virulence were found to function in phosphate homeostasis: The genes encoding plasma membrane transporters Pho84, Pho87, and Git1, the polyphosphate synthetic enzyme subunit Vtc4, and the acid phosphatase Pho100. Deletion of *PHO100* led to modest attenuation in virulence [24]; Pho100 is one of at least 3 acid phosphatases annotated in the Candida Genome Database [25].

Expression profiling of *C. albicans* in various in vitro, ex vivo and in vivo infection models consistently showed induction of *PHO84*, encoding the major high affinity proton–phosphate symporter; *PHO89,* which encodes a homolog of a high affinity *S. cerevisiae* sodium–phosphate transporter with an alkaline optimum, was also upregulated in some models [26,27,28,29,30,31]. Though these studies did not take particular note of PHO regulon components’ role in *C. albicans* interactions with the host, induction of the high-affinity phosphate transporters across a variety of infection models highlights the importance of phosphate acquisition during infection.

## 6. The Transcriptional Regulator that Controls Phosphate Homeostasis is Required for *C. albicans* Stress Resistance

A requirement for the PHO regulon transcriptional regulator, Pho4, in *C. albicans* resistance to numerous stressors was then identified in two simultaneously published screens [32,33] of two transcription factor mutant sets [34,35]. Notably, very few other transcription factors were required in multiple of these diverse forms of stress. The stressors to which *pho4* null mutant cells were hypersensitive include 2 M sorbitol (osmotic stress), 1 M NaCl and 0.6 M KCl (osmotic and cationic stress), 10 mM MnCl_2_, 5 mM FeCl_2_, 450 mM CaCl_2_, 2 mM NaAsO_2_, or 0.32 μg/mL spermidine (metal, inorganic and organic cationic stress), 5 and 10 mM H_2_O_2_ (peroxide stress), 150 and 300 uM menadione (superoxide stress), 0.6 mM S-Nitroso-L-glutathione (nitrosative stress) and pH 8 (alkaline stress) [32,33]. Pho4 was also shown to be involved in resistance to arsenic stress (an oxidative stress-inducing toxic metal taken up by phosphate transporters) [36,37]. Cells lacking Pho4 were hypersensitive to 20% serum and defective in hyphal growth in response to serum as well as in response to phagocytosis by macrophages [33]. Their survival after macrophage phagocytosis, and their ability to kill macrophages, was diminished compared to wild type [33]. In two in vivo infection models, *pho4* null mutant cells showed attenuated virulence: *Caenorhabditis elegans* that ingested *pho4* null mutant cells survived significantly longer than those fed wild type or re-integrant cells; and in a murine intravenous infection model, the outcome score (a combination of kidney fungal burden and weight loss at 3 days after infection) was significantly decreased for *pho4* null mutants [33]. In addition to the unequivocal virulence defects shown in infection models [33], a fitness defect of *pho4* null mutant cells was identified in a mouse intestine commensalism model [32]. These studies converged on the conclusion that Pho4 regulates processes by which *C. albicans* withstands a broad variety of stressors that impact the fungus during invasive disease and during commensalism.

Ikeh et al. examined which stressors induced nuclear localization of Pho4, corresponding to its active state [33]. They found that only phosphate starvation and alkaline stress, but not cationic or superoxide stress have this activating effect on Pho4 [33]. They then used reverse-transcribed RNA sequencing (RNA-Seq) to identify Pho4-controlled genes [33]. The results highlighted similarities and differences between the role of Pho4 in *C. albicans* and *S. cerevisiae*. A striking difference is that the number of *C. albicans* Pho4-regulated genes at >130 [33] is far larger than the 22 found for *S. cerevisiae* Pho4 using microarrays [38]. Similar to *S. cerevisiae*, Pho4 was found to upregulate a core set of genes involved in acquisition and storage of phosphate: the gene encoding the high-affinity phosphate transporter Pho84, transporters of glycerophosphodiesters Git1, -2, and -3, and a glycerophosphocholine phosphodiesterase Gde1. Also Pho4-regulated were several acid phosphatases (Pho100, Pho112, and Pho113) that liberate phosphate from organic compounds to make it accessible to absorption [33,39]. Additionally, genes encoding enzymes that participate in polyphosphate synthesis at the vacuolar membrane, Vtc1 and Vtc3, were found to be Pho4-induced [33]; polyphosphate is a storage polymer synthesized by vacuolar transporter chaperone (VTC) complex components Vtc1–4 [33].

The transcriptional analysis did not reveal an obvious mechanism by which Pho4 contributes to *C. albicans* stress resistance [33]. In phosphate-rich media, Pho4 normally is inactivated by phosphorylation and exits the nucleus. Intriguingly, some genes related to oxidative stress were expressed to abnormally high levels in *pho4* null mutant cells growing in phosphate rich medium: Those encoding copper-requiring superoxide dismutase enzymes Sod1, -5, and -6 [33]. This finding suggests that in wild type cells, Pho4 contributes to repression of these genes in phosphate-replete conditions, perhaps because in these conditions, cells can more readily access other mechanisms of superoxide detoxification. However, expression of *SOD3*, which encodes a fungal manganese-dependent cytosolic superoxide dismutase, and enzymatic activity of Sod1, a copper-dependent cytosolic superoxide dismutase, were decreased in *pho4* null mutant cells [33]. This was apparently due to decreased bioavailability of copper (whose total stores were not decreased in *pho4* cells), possibly because *CRD2,* encoding a copper metallothionein, was overexpressed in cells without Pho4 [33].

Two other metals were depleted in *pho4* mutant cells: they had only about 16% of the manganese and ~30% of the magnesium content of wild-type cells [33], possibly because they lacked polyphosphate as a metal chelator [33]. However, other cationic metals were not decreased in these cells [33]. Nevertheless, the metal imbalance could disturb the function of superoxide dismutases like Sod3 that require manganese [40]. Hence the contribution of the Pho4 transcriptional regulator to stress responses may be related to metal homeostasis. *pho4* mutant cells contain only ~40% of wild type cell total phosphate [33]. Lack of phosphate itself could perturb stress-buffering functions, as discussed further below.

## 7. Comparison of Pho4-Regulated Genes in Two Closely Related Yeasts, a Plant Saprobe and An Opportunistic Human Pathogen

Is the difference noted by Ikeh et al. [33], between the large *C. albicans* Pho4-regulated gene set and the limited PHO regulon of *S. cerevisiae* simply a consequence of their long evolutionary separation, is it an adaptation to the different conditions to which *S. cerevisiae* versus *C. albicans* are exposed, or both? Several groups took an ingenious approach to this question by comparing the Pho4-regulated genes of the closely related yeasts *S. cerevisiae* and *Candida glabrata,* another human-adapted yeast. Among adult patients, *C. glabrata* is now the second most frequently isolated invasive fungal pathogen [41], given selection pressure by widespread use of azole antifungal agents (to which *C. glabrata* is more resistant) among this patient population. This yeast shared a common ancestor with the plant saprobe *S. cerevisiae* only 10 million years ago and its proteins display ~75% sequence identity with those of *S. cerevisiae* [42]. Hence *C. glabrata* provides an interesting comparison of PHO regulon functions required in the lifecycle of a human commensal and pathogen, with those of plant-associated *S. cerevisiae,* that thrives on damaged fruit and persists in oak bark and leaf litter [43].

During *S. cerevisiae* cells’ response to phosphate starvation, Pho4 cooperates with another transcriptional regulator, Pho2, to control ~20 target genes [18,44,45]. Pho2 also partners with another transcription factor, Bas1, to activate genes not regulated by Pho4: Those encoding components of purine nucleotide biosynthesis that utilize the phosphate-rich intermediate phosphoribosyl pyrophosphate (PRPP); PRPP is also a substrate in pyrimidine and histidine biosynthesis [46]. *C. glabrata* Pho4 can activate transcription of most of its targets independently of cooperation with Pho2 [42,45]. The *C. glabrata* Pho4 protein is larger, comprising 533 amino acids as opposed to *S. cerevisiae* Pho4′s 313 amino acids and it regulates more genes than the *S. cerevisiae* ortholog [42,45]. Genes controlled by *S. cerevisiae* Pho4 are essentially all involved in the response to phosphate starvation and in intracellular phosphate distribution [38,44,45]. In contrast, *C. glabrata* Pho4 activates 79 genes, only 16 (~20%) of which function in phosphate homeostasis [45]. *C. glabrata* Pho4 Gene Ontology (GO) term searches for the other genes showed ~25% linked to “response to stress or chemical”, and 10% involved in “cell wall organization” [45]. Comparing Pho4-regulated genes in yeast species of distinct phylogenetic distances to *C. glabrata* and *S. cerevisiae,* with Pho4 dependence on Pho2 cooperation, the authors concluded that expansion of gene networks regulated by Pho4 correlates with its independence from Pho2 [45].

We speculate that perhaps, a single transcriptional regulator can more tightly coordinate between phosphate homeostasis and stress responses than two cooperating transcription factors. This close co-regulation could conceivably be more important for a commensal and pathogen subjected to defensive stressors by the host (from which it seeks to obtain phosphate), than for a saprotrophic yeast. Invasion of a host seems to evoke transcriptional signatures of phosphate starvation in the fungus [26,27,28,29,30,31], while dependably inducing the host to impose oxidative stress on its cells. Hence in an instance of anticipatory regulation [47], an opportunistic pathogen like *C. glabrata* may have ensured that stress responses occur promptly during host invasion by tying them to phosphate starvation responses with the same transcription factor, Pho4.

Further comparisons among fungal clades were drawn by Lev and Djordjevic who analyzed published data on genome-wide transcriptional analyses of genes controlled by Pho4 in 4 fungal species, the ascomycetes *S. cerevisiae* [38] with its close relative *C. glabrata* [45] and *C. albicans* [33], and the basidiomycete *Cryptocccus neoformans* [2]. As noted above, in *S. cerevisiae,* Pho4′s control of phosphate starvation-modulated genes requires cooperation with Pho2, unlike in the other species, while in *C. neoformans*, no Pho2 ortholog is known. These authors categorized the genes controlled by Pho4 in the human pathogens using GO terms: Genes involved in transport, lipid- and carbohydrate metabolism [2]. Additionally, in the ascomycete pathogens *C. glabrata* [45] and *C albicans*, but not in the basidiomycete *C. neoformans*, Pho4 controls genes involved in responses to stress, to chemicals and to drugs.

## 8. Cooperation of Transcriptional Regulators, Versus Control by a Single Transcription Factor, as Strategies to Coordinate Genes Required for ATP Biosynthesis

Another functional connection appears to emerge between genes regulated by Pho4 in *C. glabrata* and *C. albicans*, in which Pho4-Pho2 cooperation is diminished or absent as compared with *S. cerevisiae*, and genes regulated independently by Pho2 homologs. As mentioned, *S. cerevisiae* Pho2 (also called Bas2) controls expression of enzymes that yield adenylate, i.e., enzymes in purine and histidine biosynthesis [46], which proceeds from PRPP, a donor of high-energy phosphate groups. Hence in *S. cerevisiae*, cooperation between the 2 transcriptional regulators Pho4 and Pho2 coordinates provision of inorganic phosphate for PRPP synthesis, with purine biosynthesis [48,49], to facilitate production of the central energy- and phosphate donor of the cell, ATP. In *C. albicans*, Pho4 alone assumes some of this coordination, e.g., the gene encoding adenylate kinase, *ADK1* (controlled by Pho2 in *S. cerevisiae*), is controlled by *C. albicans* Pho4 [33]. In this way, the shift between the Pho2-, Pho4-Pho2- and Pho4- regulated genes in the saprobe compared with the human pathogens, extends to genes required to supply the metabolic precursors for ATP.

Interestingly, in addition to controlling adenylate biosynthesis genes, thereby enabling ATP production, the *C. albicans* Pho2 ortholog Grf10 contributes to hyphal growth and virulence [50,51]. We conjecture that given the central roles of adenine-based nucleotides in energy provision, metabolic enzyme cofactor synthesis, enzyme regulation through post-translational modification, second messenger production, and signaling [52], disturbed ATP production leads to wide-ranging disruption of essential functions in the cell.

## 9. The PHO Regulon Controls Transporters of Organic and Inorganic Phosphate Compounds

**Figure 1:**
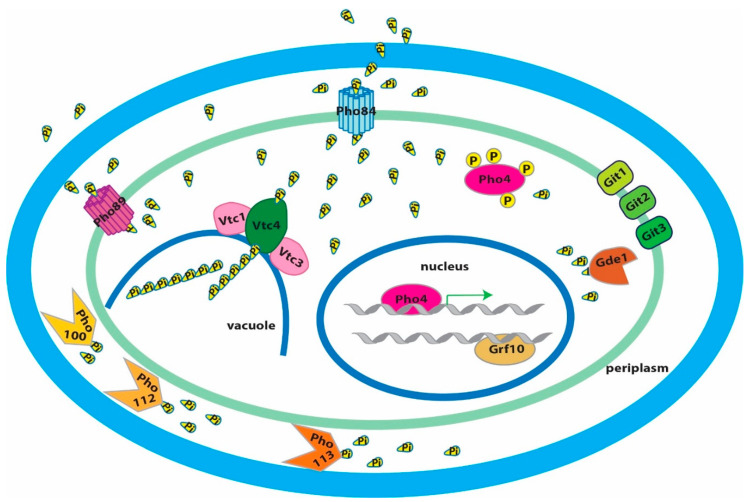
Model: *Candida albicans* phosphate (Pi) homeostasis maintenance. Under limited Pi conditions, Pi is imported by high-affinity transporters Pho84 and Pho89 whose pH optimum is in the acidic and alkaline range, respectively. Decreased Pi availability also activates the PHO regulon, whose central transcription factor Pho4 is dephosphorylated and localized to the nucleus. Independent of the interaction with the Pho2 homolog Grf10, Pho4 alone activates the expression of genes to maintain intracellular Pi homeostasis, including high-affinity Pi transporters (*PHO84* and *PHO89*), polyphosphate synthesis complex (*VTC1–4*), glycerophosphodiester transporters (*GIT1–3*), glycerophosphocholine phosphodiesterase (*GDE1*), as well as acid phosphatases (*PHO100*, *112,* and *113*).

In *C. albicans*, Pho4 controls expression of plasma membrane transporters of phosphate-containing molecules derived from plasma membrane phospholipids (glycerophosphodiesters), Git1–4 [53,54]. Comparison of these proteins with their *S. cerevisiae* orthologs showed an expanded gene family of glycerophosphocholine transporters in *C. albicans* [54]. *GIT2, 3* and *4* are aligned in tandem on chromosome 5 [54]. Git3 and to a lesser extent Git4 have important roles in uptake of glycerophosphocholine [54] while the role of Git2 has not been elucidated. *C. albicans* cells in which the entire region encoding Git2, -3, and -4 was deleted are attenuated in virulence [54], highlighting the importance of glycerophosphocholine as a phosphate source during the interaction with the host. This work also found that the cytoplasmic Gde1 glycerophosphodiesterase makes a major contribution to use of glycerophosphocholine as the sole phosphate source, presumably by hydrolyzing the ester and liberating inorganic phosphate [54]. In the same study, a *C. albicans pho4* mutant failed to upregulate *GIT3, GIT4,* or *GDE1* in response to low ambient phosphate conditions, confirming that these genes are under the control of Pho4 [54]. Figure 1 shows a cartoon of the *C. albicans* PHO regulon components discussed here.

As noted, *C. albicans PHO4* is required for resistance to alkaline stress [33]. Alkaline conditions, unlike oxidative stress, by themselves induce activation of *C. albicans* Pho4, as determined from its localization to the nucleus [33]. Since multiple homeostatic mechanisms maintain the pH of human blood and tissues between 7.35 and 7.45, *C. albicans* undergoes alkaline stress during invasive disease. Lev and Djordjevic argue that in the host, alkaline stress causes phosphate starvation responses despite inorganic phosphate concentrations in the 1–2 mM range, because the major high-affinity phosphate–proton symporter Pho84 requires a proton gradient across the fungal plasma membrane [2]. Given its mechanism of phosphate transport, by which protonation of a conserved amino acid in the transmembrane channel (Asp^358^ in *S. cerevisiae* [55]) is a condition for a conformational change that enables phosphate transport [55], this rationale seems highly plausible. Interestingly, the other known phosphate transporters in *C. albicans* appear unable to provide sufficient phosphate in alkaline pH and moderate phosphate concentrations: Pho89 is a high-affinity transporter with an alkaline optimum in *S. cerevisiae* and in that organism is controlled by the stress responsive calcineurin-dependent transcription factor Crz1, not by Pho4/Pho2 [56]. The *C. albicans* genome encodes homologs of at least 2 other *S. cerevisiae* low-affinity phosphate transporters that are active in high ambient phosphate, Pho87 and Pho90 [25,57].

We speculate that, as in other fungi like *S. cerevisiae*, Pho84 is the critical plasma membrane transporter in *C. albicans* and can be only partially substituted by Pho89 and the low-affinity transporters, because the fungus is not fully adapted to conditions prevailing during invasive disease in the host. In line with the rationale proposed by Lev and Djordjevic [2], alkaline hypersensitivity of the *pho4* null mutant analyzed by Ikeh et al. [33] may therefore be attributable to these cells’ impaired *PHO84* expression. The circumstances that *C. albicans* apparently relies on a phosphate–proton symporter whose pH optimum is 4–5, not on the Na^+^-phosphate symporter Pho89 (which has an alkaline optimum), while the pH of healthy human tissue and blood induces alkaline stress, suggest that invasive candidiasis is an accidental incursion for which the fungus is evolutionarily ill-prepared.

## 10. A Pho84 Function Activates *C. albicans* TORC1, and TORC1 Modulates the PHO Regulon

A first characterization of *C. albicans* Pho84 was undertaken when its role in activating signaling of Target of Rapamycin Complex 1 (TORC1) was identified in a screen for rapamycin hypersensitive *C. albicans* mariner transposon mutants [15]. TORC1 signaling activity was found to depend both on the phosphate availability in ambient media, and on the presence of Pho84, which appears to signal to TORC1 through the upstream small GTPase Gtr1 [15]. In *S. cerevisiae*, Pho84 is known to have a signaling role to protein kinase A (PKA) that is genetically and chemically separable from its phosphate transport activity [55,58,59,60]; whether in *C. albicans*, Pho84 has such a “transceptor” [55,59,61] role towards PKA or TORC1 remains to be experimentally established. TORC1 integrates eukaryotic cells’ responses to nutrient availability and absence or presence of stressors as the major regulatory module, inducing growth and proliferation in favorable conditions of ample nutrients and absent stress, or growth arrest and stress responses during starvation and stress exposure [62,63,64]. Classically, TORC1 is known to respond to nitrogen sources [65] and this has been experimentally determined to be true in *C. albicans* as well [66]. It was shown that phosphate availability is integrated into the TORC1 signal of *C. albicans* and *S. cerevisiae* [15]: an unsurprising finding given that phosphate is a critical component of large cellular structures like chromosomes, ribosomes, mRNA and membranes, and nitrogen and carbon source repletion is not adequate for anabolic processes like DNA replication or ribosome biogenesis in the absence of sufficient phosphate [67].

TORC1 co-regulates expression of genes required for nitrogen source acquisition like the ammonium transporter Mep2 during nitrogen-starvation versus -refeeding [68]. A role for the Tor1 kinase was also shown in phosphate acquisition, for downmodulating *PHO84* expression during phosphate refeeding [15]. Similarly, TORC1 artificially activated by ectopic *GTR1* overexpression downregulates secreted acid phosphatase during phosphate starvation that is normally induced by the PHO regulon [15]. TORC1 signaling therefore acts on the PHO regulon to fine-tune the fungal cell’s resource expenditures on proteins deployed for phosphate acquisition.

Intact *C. albicans* TORC1 signaling regulates morphogenesis and other cellular processes that are active during infection [69,70]. The potent *C. albicans* TORC1 inhibitor rapamycin is cidal for the fungus [71,72]. Rapamycin is unsuitable as an antifungal agent because it is also highly active against human TORC1, resulting in immunosuppression at low doses and multiple toxicities at higher ones [73,74]. For this reason, it was considered that inhibiting Pho84, which has no human homolog, might be an indirect, nontoxic route to inhibiting *C. albicans* TORC1 [15]. Small molecules phosphonoacetic acid and phosphonoformic acid (foscarnet), an antiviral agent in clinical use, inhibit Pho84 in *S. cerevisiae* [59] and *C. albicans* and induce downregulation of TORC1 signaling [15]. They also potentiate the antifungal activity of amphotericin B and micafungin against wild type *C. albicans*, recapitulating the hypersensitivity phenotypes of *pho84* mutant cells against these drugs [15]. Amphotericin and micafungin represent 2 major classes of antifungal agents, the polyenes and echinocandins. Foscarnet itself has significant toxicities but as a proof of concept can inspire the search for other Pho84 inhibitors with fewer off-target effects, which could potentiate these antifungal agents.

## 11. Defects in Virulence and Oxidative Stress Resistance of Cells Lacking Pho84

Cells lacking Pho84 show virulence attenuation in murine and *Drosophila* models of infection [75]. These cells are defective in hyphal growth and hypersensitive to killing by human whole blood and by neutrophils [75]. Releasing superoxide anion in the oxidative burst is a major antimicrobial killing mechanism of neutrophils; *pho84* null mutant cells were not hypersensitive to neutrophils from a patient with chronic granulomatous disease which lack the capacity to generate an oxidative burst [75]. Accordingly cells lacking Pho84 poorly tolerate pharmacologic superoxide generators compared to wild type [75]. Measurement of intracellular reactive oxygen species (ROS) showed that both during extrinsic oxidative stress, and in unstressed normal growth conditions, *pho84* null mutant cells contain more ROS than wild type [75]. A major signaling system that activates survival responses to oxidative stress, the HOG pathway, is hyperactive in *pho84* null mutant cells exposed to peroxide; hence a defect not in signaling, but in the execution of oxidative stress management systems is apparently responsible for the accumulation of ROS and hypersensitivity to extrinsic oxidative stress shown by *pho84* null mutant cells [75].

Polyphosphate acts as an antioxidant in other organisms [76]. In *S. cerevisiae*, it is synthesized on the vacuolar membrane and translocated into the vacuolar lumen by the vacuolar transport complex (VTC) whose catalytic subunit Vtc4, transfers the gamma phosphate of ATP onto a growing phosphate anhydride chain [77,78]. It was considered that *C. albicans* cells defective in phosphate acquisition might be hypersensitive to oxidative stress because they lack an antioxidant activity of polyphosphate [33]. However, Ikeh et al. did not find hypersensitivity to stressors, including superoxide stress, in *C. albicans* mutants lacking the catalytic (Vtc4) or a regulatory component (Vtc1) of the VTC [33]. Hence a potential contribution of polyphosphate to oxidative stress management in *C. albicans* remains undefined.

Expression of the unique manganese-containing cytosolic superoxide dismutase, Sod3 [40,79], is low at the mRNA level in *pho4* mutants [33], as it is at the protein level in *pho84* null mutant cells [75]. These findings are mutually consistent because *PHO84* expression depends on Pho4 activity [33], so that cells without Pho4 are expected to show phenotypes corresponding to lack of Pho84. Sod3 expression was found to represent a link between Pho84 and TORC1: Cells lacking Pho84, in which TORC1 is artificially activated by *GTR1* overexpression, recover Sod3 expression and their intracellular ROS increase is largely rescued [75]. Conversely, overexpression of *SOD3* from a heterologous promoter significantly—but not completely—rescues oxidative stress hypersensitivity of *pho84* null mutant cells [75]. Neither *GTR1* nor *SOD3* overexpression rescues *pho84* null mutant cells’ hypersensitivity to whole blood and neutrophils, though, indicating that defective TORC1 signaling and subsequent low Sod3 levels are one, but not the only mechanism of these cells’ hypersensitivity to ex vivo host defense models [75].

## 12. Cell Wall Stress Hypersensitivity of *C. albicans* Cells Lacking Pho84

What other molecular alterations in *pho84* null mutant cells are responsible for their hypersensitivity to host immune mechanisms? Another possible weakness of these cells is their defective cell wall [80]. Their outer phosphomannan cell wall layer is thinner, they contain less chitin and beta-1,6-glucan, and they are hypersensitive to cell wall stress [80]. The cell wall stressors to which cells lacking Pho84 are susceptible are varied and include beta-1,3-glucan synthase inhibition by the echinocandin micafungin, chitin synthase inhibition by nikkomycin, heat, and disruption of chitin-glucan bonds by Congo red [80]. Hypersensitivity of *C. albicans* wild type and *pho84* null mutant cells to pharmacologic cell wall stressors is inversely related to their phosphate repletion state [80]. This hypersensitivity corresponds to deficient cell wall stress signaling as assayed by the phosphorylation state of the cell wall integrity pathway MAP kinase Mkc1 [80]. Mechanical weakness of the cell wall might contribute to *pho84* null mutant cells’ inability to withstand neutrophil hydrolytic enzymes [81]. Cell wall integrity defects of *pho84* null mutant cells cannot be rescued by artificial TORC1 activation [80], suggesting they may be independent of these cells’ TORC1 signaling perturbation. 

## 13. *pho84* Null Mutant Cells Lack Nucleotide Sugars, Substrates of Cell Wall Biosynthetic Enzymes

Metabolomics experiments intimated how lack of *C. albicans* Pho84 could result in cell wall defects. The substrates of biosynthetic enzymes that produce the major structural cell wall polysaccharides, beta-1,3- and beta-1,6-glucan and chitin, are the nucleotide sugars uridine-diphosphate (UDP)-glucose and UDP-N-Acetylglucosamine, respectively. Both these substrates, as well as their phosphoric precursor uridine-triphosphate (UTP), were significantly diminished in *pho84* null mutant cells recovering from phosphate starvation, compared with wild type cells [80]. Similarly, GTP, a precursor in the production of GDP-mannose required in cell wall phosphomannan biosynthesis, was drastically diminished in these cells, as were the other nucleotides including ATP [80]. Strikingly, in nucleotide and nucleotide sugar biosynthetic pathways, the intermediates before a phosphorylation step accumulated, while those after it were diminished in cells lacking Pho84 [80]. This finding suggests that lack of the phosphate provided by ATP also slows the reaction rate of kinases in pyrimidine and purine nucleotide synthesis [80]. The result is that substrate concentrations for beta-1,3- and beta-1,6-glucan synthases, as well as for chitin synthases, are low in cells lacking Pho84 [80]. The polysaccharide products of these enzymes provide the structural stability and the shape of the cell wall.

Lack of Pho84 potentiated *C. albicans* growth inhibition arising from pharmacologic or genetic perturbation of glucan and chitin synthase activity [80]. It also blocked normal compensatory stimulation of chitin production when beta-1,6-glucan synthase is genetically depleted [80,82]. A simple model emerges from these findings: lack of their nucleotide sugar substrates slows the reaction rate of enzymes that synthesize cell wall polysaccharides in cells without Pho84, leading to a dearth of cell wall structural stabilizers and a weakened cell wall. Potentiation of micafungin, a beta-1,3-glucan synthase inhibitor, by the Pho84 inhibitor foscarnet may presage an antifungal combination in which cell wall biosynthesis is inhibited at 2 successive steps, at nucleotide sugar biosynthesis and at glucan or chitin synthesis. A paradigm for deploying inhibitors of successive steps in a crucial microbial biosynthetic process to potentiate the effect of each, is inhibition of tetrahydrofolate biosynthesis by the combination of trimethoprim with sulfamethoxazole (Bactrim or Cotrimoxazole), an antibiotic that is still essential after >4 decades [83]. Perhaps this paradigm could also be applied successfully in antifungal development.

## 14. Lack of ATP Impacts *C. albicans* Carbon Metabolism

Other critical pathways in central metabolism are significantly affected by lack of *C. albicans* Pho84: glycolysis, the citrate (TCA) cycle, and the pentose phosphate pathway [80]. Given the need for 2 molecules of ATP for every glucose molecule in the preparatory phase of glycolysis, and the utilization of an inorganic phosphate in the 6th reaction of glycolysis [52], slowing of glycolysis in conditions of low ATP and low free cytosolic phosphate that prevail in *pho84* null mutant cells [15] seems intuitive. Inorganic phosphate is needed to generate ATP, whether in the cytosol or in the mitochondrial matrix where it is a required substrate of ATP synthase; the essential role of mitochondrial phosphate import was recently shown when a novel antifungal agent was characterized whose fungicidal activity occurs through blocking a mitochondrial phosphate pump [84]. Similarly, lack of inorganic phosphate in the mitochondrial matrix might impede generation of ATP by oxidative phosphorylation. In fact, NADH, the electron carrier at the entry point into oxidative phosphorylation, is elevated in *pho84* null mutant cells compared to wild type, while its oxidized redox partner NAD is depleted [80], suggesting a block in a major cellular consumption point of NADH, oxidative phosphorylation.

Lack of ATP may introduce further inefficiencies of carbon catabolism, by impeding biosynthesis of coenzyme A [85] which is required for entry of 2-carbon units into the citric acid cycle and is decreased in *pho84* null mutant cells [80], or by slowing entry into the pentose phosphate pathway through diminished glucose phosphorylation, as these cells contain less ribose-phosphate [80].

Hence to generate ATP, both inorganic phosphate, and the energy from oxidation of carbon sources to create the third, high-energy phosphate bond are necessary. To utilize these carbon sources, upfront investment of ATP is required to channel carbon sources into glycolysis, the pentose phosphate cycle, and the citric acid cycle. We speculate that lack of phosphate may feed forward to further impede ATP synthesis, when the fungal cell is unable to utilize the energy potential of its carbon sources because the upfront investment cannot be made.

## 15. Metabolites with Roles in Oxidative Stress Management are Decreased in *C. albicans* Cells Lacking Pho84

Whether phosphate starvation-related inefficiency of oxidative phosphorylation in cells lacking Pho84 increases the constitutive generation of ROS during this process [52] needs to be experimentally tested. However, compounds that participate in detoxifying ROS in other species, are diminished in these cells [80]: thiamine pyrophosphate [86] and ascorbic acid [87].

Purine metabolism, which is disrupted in *pho84* null mutant cells, is one of the pathways leading into thiamine pyrophosphate biosynthesis. Thiamine when added exogenously protected *E. coli* against intracellular superoxide anions induced by paraquat, and it diminished induction of oxidative stress response genes [88]. These authors speculated that thiamine might act as a direct scavenger of superoxide anions [88]. Biosynthetic enzymes of this coenzyme are induced during oxidative stress in *S. cerevisiae* in connection with Hog1 and Yap1 [86] while exogenous administration of thiamine protects *S. cerevisiae* cells exposed to peroxide stress [89]. A role of thiamine in *C. albicans* stress responses has not yet been examined.

**Figure 2:**
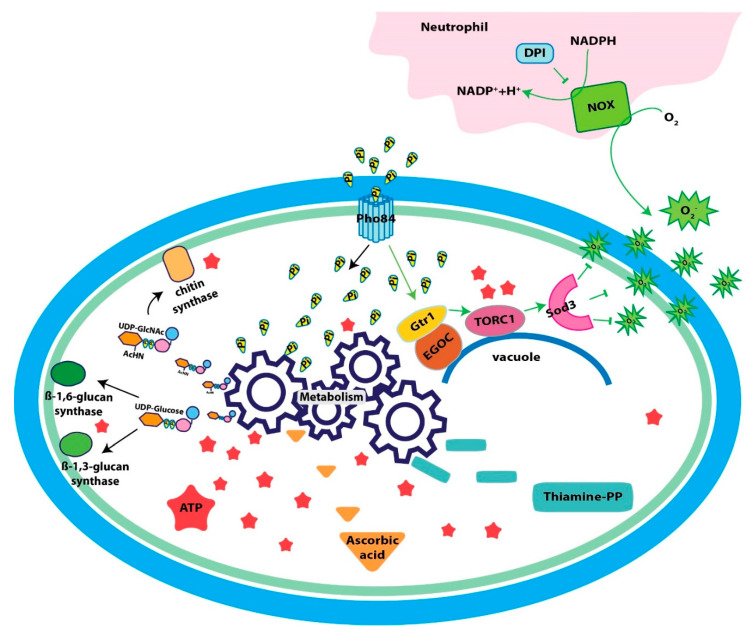
Model: Pho84 contributes to *C. albicans* TORC1 signaling, metabolism, cell wall integrity and oxidative stress defense. The high-affinity inorganic phosphate (Pi) transporter Pho84 plays a significant role in cellular import of Pi. Cytosolic and mitochondrial Pi becomes available to metabolic processes that produce substrates for cell wall construction enzymes (nucleotide sugars UDP-glucose and UDP-N-Acetylglucosamine, GlcNAc), the energy currency ATP, and co-factors with possible roles in detoxifying reactive oxygen species (ROS), i.e., thiamine pyrophosphate and erythro-ascorbic acid. Pho84 also contributes to oxidative stress management by activating TORC1 through Gtr1. TORC1 induces the expression of superoxide dismutase Sod3, contributing to *C. albicans* tolerance of ROS produced by host immune cells.

In addition to being the substrate of beta-1,3- and beta-1,6- glucan synthases, the nucleotide sugar UDP-glucose is a precursor in the biosynthesis of erythro-ascorbic acid, an analog of Vitamin C synthesized by *S. cerevisiae* and *C. albicans* [87,90]. Presumably it is erythro-ascorbic acid whose LC-MS/MS profile in *C. albicans* metabolome extracts was interpreted as ascorbic acid, and whose content was drastically reduced in *pho84* null mutant cells recovering from phosphate starvation in excess phosphate [80]. The significantly diminished UDP-glucose content of cells lacking Pho84 may therefore determine not only their cell wall integrity defects, but also affect their ability to produce erythro-ascorbic acid as a reducing agent in managing intracellular ROS [87,90]. *C. albicans* cells engineered to lack the final enzyme in the erythro-ascorbic acid biosynthetic pathway are defective in hyphal growth and attenuated in virulence in an intravenous mouse infection model [90]; these investigators surmised that erythro-ascorbic acid may be an important antioxidant in the host interaction to enhance survival of the fungus during host-imposed oxidative stress [90]. Figure 2 shows a selection of the Pho84-connected processes that contribute to tolerance of oxidative stress and cell wall stress in *C. albicans*.

## 16. Perturbation of Phosphate Homeostasis may be Clinically Useful

Given the essential role of phosphate in all core processes of the cell, pharmacologically inhibiting its acquisition might impact growth and virulence of *Candida* species in therapeutically useful ways. A critical advantage of phosphate homeostasis as a drug target is that this process shows 2 fundamental differences between fungi and humans. Firstly, most human phosphate homeostasis disturbances relate to failure to sufficiently excrete excess phosphate, e.g., in renal insufficiency (except during starvation or in rare genetic conditions) [91]. This is because we inevitably acquire substantial amounts of phosphate with the plant- or animal-based foods that provide enough protein. Secondly, human phosphate transporters must function in conditions of neutral or mildly alkaline pH, while in fungi, a proton-phosphate symporter whose optimum is acidic is the most efficient transporter. This proton-phosphate symporter in *C. albicans* is Pho84 and it has no human homolog. In the basidiomycete pathogen *Cryptococcus neoformans*, virulence is attenuated when the 3 high-affinity Pi transporters at the cell surface are mutated [92]. Given the essentiality of phosphate in cellular functions, it is striking that *C. albicans* has not evolved more redundancy for Pho84 activity: among at least 3 others, loss of this single plasma membrane phosphate importer is sufficient to affect virulence [75].

An efficient inhibitor of Pho84 hence not only may directly slow growth by diminishing phosphate supplies of the fungal cell and by inhibiting TORC1 signaling, but could also block oxidative stress management, attenuating virulence. Furthermore, by depleting the cell of substrates for cell wall biosynthetic enzymes, it could weaken the cell wall and potentiate the activity of drugs that inhibit these enzymes.

Much remains to be discovered in how *Candida* cells regulate the acquisition and distribution of phosphate. Recent exciting work in other fungi implicates inositol pyrophosphates in regulating elements of the PHO regulon through N-terminal SPX domains of these proteins [93]. Delineating differences between *S. cerevisiae* and *Candida* PHO regulons will shed further light on the specific phosphate-related requirements of a human commensal and opportunist. The role of ATP depletion in multiple virulence-related cellular processes awaits further analysis. The finding that *pho84* null cells’ walls contain less of the polysaccharides whose synthesis requires nucleotide sugar substrates, raises the question how the cell allocates scarce phosphate supplies to each cellular process. Does it prioritize translation over cell wall construction? Along the path of these coming discoveries, novel drug targets are likely to appear given the difference of fungal and human cellular phosphate management. As more fungal pathogens like *Candida auris* emerge, more questions will arise. Some of the answers may contribute to medical advances.
